# Integrative analysis and imputation of multiple data streams via deep Gaussian processes

**DOI:** 10.1093/bioadv/vbaf305

**Published:** 2025-11-27

**Authors:** Ali A Septiandri, Deyu Ming, Francisco Alejandro DiazDelaO, Takoua Jendoubi, Samiran Ray

**Affiliations:** Department of Statistical Science, University College London, London WC1E 7HB, United Kingdom; School of Management, University College London, London WC1E 6BT, United Kingdom; Clinical Operational Research Unit, University College London, London WC1H 0BT, United Kingdom; Department of Statistical Science, University College London, London WC1E 7HB, United Kingdom; Paediatric Intensive Care Unit, Great Ormond Street Hospital For Children NHS Foundation Trust, London WC1N 3BH, United Kingdom

## Abstract

**Motivation:**

Healthcare data, particularly in critical care settings, presents three key challenges for analysis. First, physiological measurements come from different sources but are inherently related. Yet, traditional methods often treat each measurement type independently, losing valuable information about their relationships. Second, clinical measurements are collected at irregular intervals, and these sampling times can carry clinical meaning. Finally, the prevalence of missing values. Whilst several imputation methods exist to tackle this common problem, they often fail to address the temporal nature of the data or provide estimates of uncertainty in their predictions.

**Results:**

We propose using deep Gaussian process emulation with stochastic imputation, a methodology initially conceived to deal with computationally expensive models and uncertainty quantification, to solve the problem of handling missing values that naturally occur in critical care data. This method leverages longitudinal and cross-sectional information and provides uncertainty estimation for the imputed values. Our evaluation of a clinical dataset shows that the proposed method performs better than conventional methods, such as multiple imputations with chained equations (MICE), last-known value imputation, and individually fitted Gaussian processes (GPs).

**Availability and implementation:**

The source code of the experiments is freely available at: https://github.com/aliakbars/dgpsi-picu.

## 1 Introduction

One of the main challenges in analysing healthcare data is that they are usually collected from multiple measurement streams. A patient’s medical record may include data from different sources, such as medical histories, laboratory tests, and imaging studies (Johnson *et al.* 2016). Integrating and analysing the data can thus be challenging, as the various data sources may use different formats, units, and scales. For example, a patient’s CO_2_ level may be measured breath by breath directly from a ventilator circuit, but their albumin levels are measured daily from blood sample tests.

The difference in sampling frequency for data from multiple sources (Groenwold 2020) relates to the cost to both the patient and the observer. For example, non-invasive measurements are sampled frequently, whereas invasive measurements, which require technically difficult procedures, are sampled less frequently. Blood sampling comes at a cost to the patient in loss of blood volume—while this will be relatively small for adults, this can be significant enough for children that they may need blood transfusions (François *et al.* 2022, Siegal *et al.* 2023).

With the difference in sampling frequency, aligning these multiple streams of data will result in missing values. Simply removing missing values and performing a complete case analysis would not suffice, since useful observations might be lost (Vesin *et al.* 2013). Additionally, there is the problem of informative sampling, where one might observe an extended period of intervals because the patient is getting better, making the clinicians reduce the frequency of monitoring (Che *et al.* 2018). These circumstances make it challenging to assess a patient’s health and make informed decisions accurately. As the sampling frequency is, by nature, informative, it will be more difficult to detect subtle changes in unobserved variables retrospectively.

Current practices for handling missing values in healthcare data often prioritize simplicity over complexity. A common approach is using the last-known value imputation, also known as the last-observation-carried-forward (LOCF) method, which fills in missing values by extending the most recent available measurement for a specified time period (Siddiqui and Ali 1998). While this method is straightforward, it overlooks the correlation between covariates. On the other hand, multiple imputation by chained equations (MICE), another widely used method, uses cross-sectional information between covariates but treats each observation independently (Rubin 1987, Tsvetanova *et al.* 2021).

More recently, deep neural networks have become increasingly popular for handling missing values in healthcare data (Lipton *et al.* 2016, Che *et al.* 2018, Cao *et al.* 2018, Yıldız *et al.* 2022). However, these methods have a limitation: they typically do not provide estimates of uncertainty in their predictions. This can be a problem in healthcare data analysis, where medical observations and interpretations inherently contain uncertainty, which may come from measurement error, inherent noise in the signal, or the use of surrogate markers. When this uncertainty in the input data is not accounted for, it can lead to unreliable model predictions (Cabitza *et al.* 2017).

This study aims to tackle the problem of handling missing values in multivariate time series data by leveraging both longitudinal and cross-sectional information. We use a deep Gaussian process (GP) model with stochastic imputation (Ming and Guillas 2021, Ming *et al.* 2023) where time is the input to predict the target variable through covariates in the model’s latent layer. By fitting the model jointly, the available information can be used as leverage to impute missing values stochastically. Moreover, this method comes with uncertainty estimation. This approach is evaluated against a baseline method that fits individual GPs to covariates using complete case analysis for model training and subsequent imputation.

A deep Gaussian process model is a hierarchical structure of GP nodes organized in layers to represent latent variables (Damianou and Lawrence 2013). Each node receives input from the previous layer and produces output that serves as input for the next layer. The observed data points appear at the final layer of this hierarchy. While single-layer GP models are limited by the kernel function used, which can be highly parametrized to learn complex data patterns, a deep GP model learns them non-parametrically via the hierarchy, thus having fewer hyperparameters to optimize (Salimbeni and Deisenroth 2017). Due to their ability to provide uncertainty estimates, deep GP models have applications in real-life domains, including aero-propulsion system simulation (Biggio *et al.* 2021), crop yield prediction from remote sensing data (You *et al.* 2017), and uncertainty estimation in electronic health records (Li *et al.* 2021).

The remainder of the paper is structured as follows. The clinical problem that motivated this research is explained in Section 2. GPs and deep GPs are reviewed in Section 3, where the methodological approach and alternative techniques for handling missing values in multivariate time series data are described. The proposed deep GP using stochastic imputation is validated by applying it to a clinical case study in Section 4. Finally, findings and future directions are summarized in Section 5.

## 2 Motivation: clinical problem

Missing values are a common challenge in healthcare datasets, arising from sources such as incomplete patient forms, survey non-responses (Penny and Atkinson 2012), and technical glitches during data collection (Zhang and Koru 2020). These missing values manifest within the data and affect study outcomes and statistical validity. Therefore, choosing appropriate methodological approaches to tackle this problem is crucial.

In critical care medicine, missing values are also a result of irregular and informative sampling (Groenwold 2020). To provide some context, clinicians in critical care monitor deviations from the expected arterial acidity (pH) range to gain insights into respiratory function, electrolyte balance, and the underlying diseases of the patients (Sirker *et al.* 2002). When pH deviates from normal ranges, either through acidosis (pH <7.3) or alkalosis (pH >7.5), it could disrupt vital biochemical processes and overall equilibrium, with studies showing that blood pH levels are associated with the mortality rate (Jung *et al.* 2011, Rodríguez-Villar *et al.* 2021) and neurological recovery in cases of cardiopulmonary resuscitation (Shin *et al.* 2017).

One way to monitor and model the pH level is the Stewart-Fencl approach (Stewart 1978, 1983, Fencl and Leith 1993). This approach identifies three independent variables that determine pH. The first variable is carbon dioxide (CO_2_), a major source of acid in the body that can be continuously monitored using modern bedside equipment. The second and third variables are strong ion differences (e.g. Na${\;}^{+}$, K${\;}^{+}$, Cl${\;}^{-}$) and weak acids (e.g. albumin, lactate, urea, phosphate), respectively. These components require blood tests for measurement, which are performed less frequently due to their invasive nature (Barie 2004).

While CO_2_ is also measured through capnography as a surrogate, known as end-tidal CO_2_ (ETCO_2_), the main interest is in blood CO_2_ levels because they directly influence pH. In various respiratory conditions (such as asthma and COPD), the CO_2_ in the blood does not equilibrate with CO_2_ in the lungs. This creates a measurable gap between blood and alveolar CO_2_ levels, which can also be informative (Anderson and Breen 2000, Lee *et al.* 2024).

**Figure 1.**
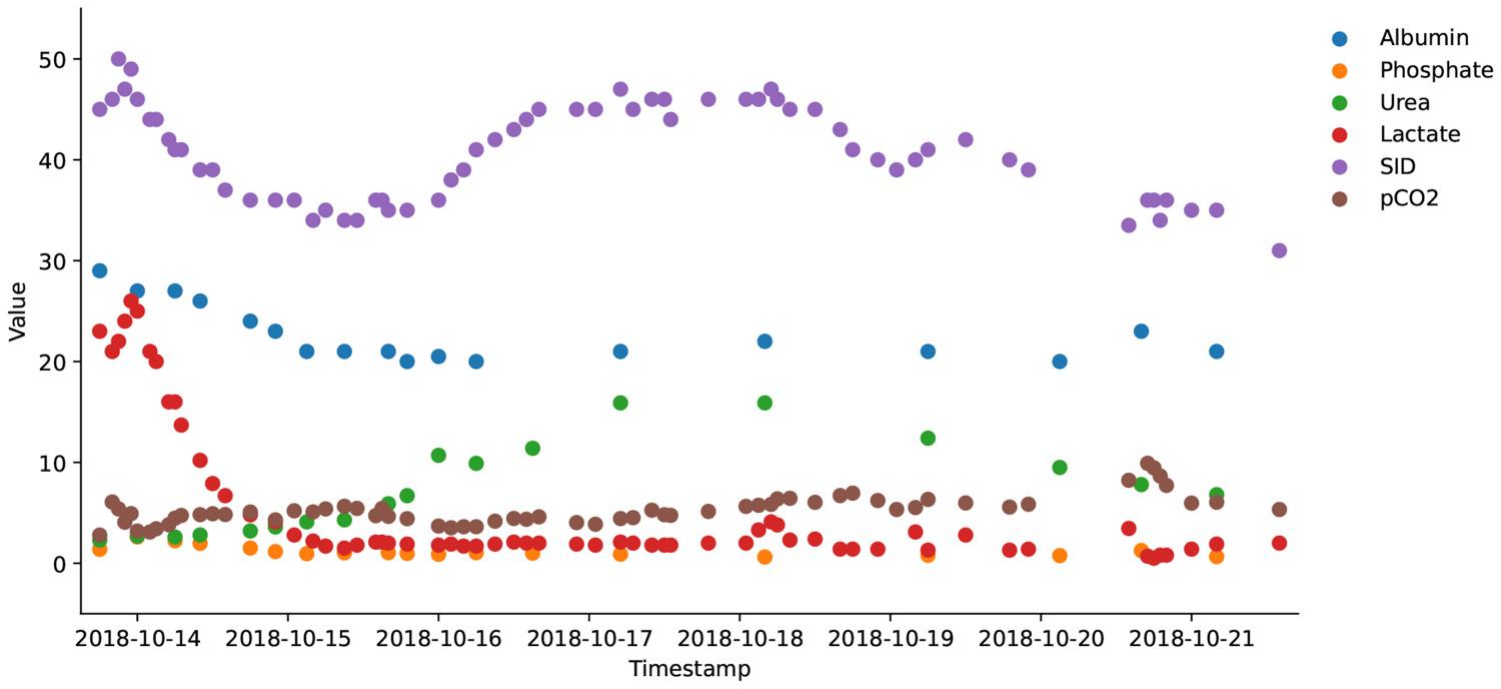
Irregular sampling of six measurements of a sample patient. The pCO_2_, strong ion differences (SID), and lactate are measured from bedside monitoring, while albumin, phosphate, and urea levels are obtained from blood tests.

This disparity in measurement frequency creates a pattern of irregular sampling, where data collection occurs at inconsistent intervals. The resulting gaps in data collection lead to missing values, particularly for parameters requiring blood tests, as illustrated in Fig. 1. Consequently, the irregular nature of these measurements complicates the application of standard statistical techniques to analyse and interpret the data.

On the other hand, informative sampling occurs when data point selection is influenced by factors related to clinical hypotheses. For instance, clinicians might refrain from collecting data when a patient’s health improves and vice versa (Che *et al.* 2018). In this context, both irregular and informative sampling scenarios fall under the Missing Not at Random (MNAR) category, as the missingness is not random but associated with unobserved data or specific conditions and the missingness carries information (Little and Rubin 2019).

This study addresses the challenge of missing values in critical care data, which can impact patient outcome predictions (Vesin *et al.* 2013), for example, in predicting in-hospital and 30-day mortality (Sharafoddini *et al.* 2019). Rather than relying on complete-case analysis, which risks losing valuable information, this work proposes using deep GPs for data imputation. This approach leverages both cross-sectional and longitudinal information from patient records.

Following the Stewart-Fencl approach, the analysis is structured with arterial pH as the dependent variable. Relevant covariates are constructed to identify factors causing pH deviations. The proposed method not only imputes missing values in the covariates but also quantifies the uncertainty associated with these imputations, providing a more comprehensive understanding of the data’s reliability. Although pH is primarily used to evaluate acid-base status, other covariates provide critical information on metabolic status relevant to patient care in critical care settings (Gattinoni *et al.* 2017).

This study focuses on its application in critical care medicine because of its clinical importance, but the proposed method applies to different scenarios with similar conditions. For example, missing values are also found in human activity recognition from multiple sensor streams (Jain *et al.* 2022), sleep disorder diagnoses using electroencephalogram (EEG) (Lee *et al.* 2021), and hepatocellular carcinoma (Han *et al.* 2021).

## 3 Methodology

### 3.1 Gaussian processes

Let $X\in R^{N\times D}$ represent a *D*-dimensional input with *N* observed data points and $Y\in R^{N}$ be the corresponding outputs. Then, the GP model assumes that Y follows a multivariate Gaussian distribution $Y∼N(\mu ,\sigma^{2}R(X))$, where $\mu \in R^{N}$ is the mean vector, $\sigma^{2}$ is the scale parameter, and $R(X)\in R^{N\times N}$ is the correlation matrix. Cell *ij* in the matrix R(X) is specified by $k(X_{i*},X_{j*})+\eta 1_{\left\{X_{i*}=X_{j*}\right\}}$, where k(·,·) is a given kernel function with η being the nugget term and $1_{\left\{\cdot \right\}}$ being the indicator function. In this study, we consider Gaussian processes with zero means, i.e. μ=0 and kernel functions with the multiplicative form: $k(X_{i*},X_{j*})=\prod_{d=1}^{D}k_{d}(X_{id},X_{jd})$ where $k_{d}(X_{id},X_{jd})=k_{d}(|X_{id}-X_{jd}|)$ is a one-dimensional stationary and isotropic kernel function, e.g., squared exponential kernel function (Rasmussen and Williams 2006), for the *d*-th input dimension.

The hyperparameters $\sigma^{2}$, η, and those contained in k(·,·) are typically estimated using maximum likelihood or maximum a posteriori (Rasmussen and Williams 2006). Given estimated GP hyperparameters, the realizations of input $x={\left(x_{1*}^{T},\ldots ,x_{N*}^{T}\right)}^{T}$, and output $y={\left(y_{1},\ldots ,y_{N}\right)}^{T}$, then the posterior predictive distribution of output $Y_{0}$ at a new input position $x_{0}\in R^{1\times D}$ follows a Gaussian distribution with mean $\mu_{0}$ and variance $\sigma_{0}^{2}$ given by:


(1) $$\mu_{0}=r{\left(x_{0}\right)}^{T}R{\left(x\right)}^{-1}y$$

(2) $$\sigma_{0}^{2}=\sigma^{2}(1+\eta -r{\left(x_{0}\right)}^{T}R{\left(x\right)}^{-1}r(x_{0}))$$

where $r(x_{0})={\left[k(x_{0},x_{1*}),\ldots ,k(x_{0},x_{N*})\right]}^{T}$.

A GP model can be used as a smoothing function for irregularly sampled signals through the predicted mean function of a time series. Thus, GPs have been used to model electronic health records (Lasko *et al.* 2013), wearable sensor data for e-health (Clifton *et al.* 2013), gene expression data (Gao *et al.* 2008, Kirk and Stumpf 2009, Liu *et al.* 2010), and quantitative traits (Vanhatalo *et al.* 2019, Arjas *et al.* 2020).

### 3.2 Linked Gaussian processes

Consider *P* GP models $GP_{1}^{\left(p\right)}$ for p=1,…,P, where *P* is the total number of output dimensions of computer models with $N_{p}$ sets of $D_{p}$-dimensional input ($X_{p}\in R^{N_{p}\times D_{p}}$) and produces $N_{p}$ sets of one-dimensional output ($W_{p}\in R^{N_{p}\times 1}$). In the Stewart–Fencl approach, this multi-output GP model can be interpreted as using time as a shared input variable, but with differing numbers of training points, and predicting covariates as outputs. Let $GP_{2}$ be another GP model with *M* sets of *P*-dimensional input ($W\in R^{M\times P}$) and one-dimensional output ($Y\in R^{M\times 1}$), where the *P* features (i.e. dimensions) in W correspond to the *P*-dimensional outputs produced by ${\left\{GP_{1}^{\left(p\right)}\right\}}_{p=1,\ldots ,P}$. A linked GP (LGP) is then created when we feed the predictions from ${\left\{GP_{1}^{\left(p\right)}\right\}}_{p=1,\ldots ,P}$ into the input of $GP_{2}$.

**Figure 2.**
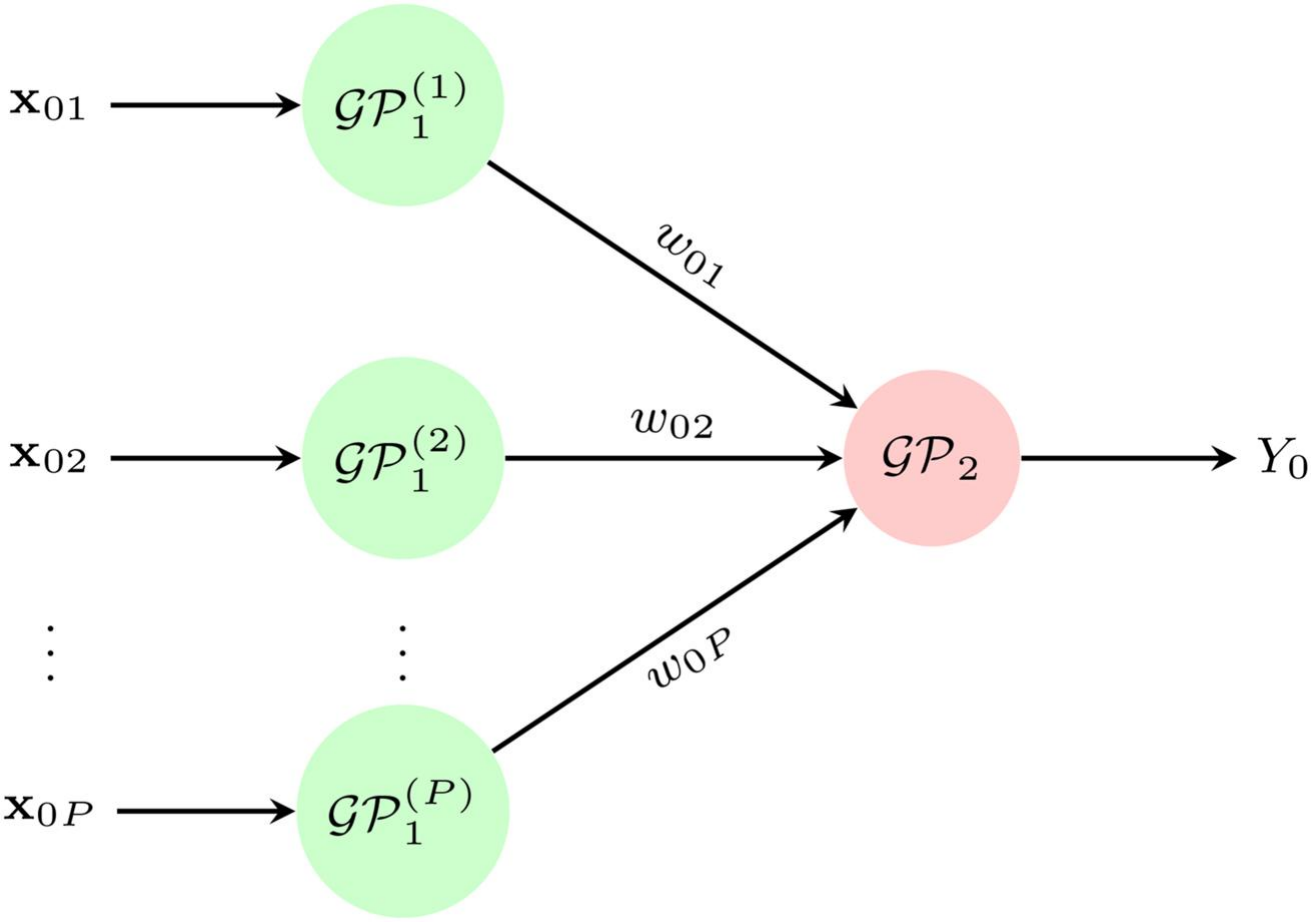
A two-layered linked Gaussian process model. In the Stewart–Fencl approach, the first layer can be seen as using time as the input variable and predicting covariates as outputs, where the outputs are conditionally independent with respect to X. A special case arises when all models in the first layer share time as a common input variable with an equal number of training points. The second GP layer then takes the outputs from the first layer as its inputs to model pH as the output variable. This model corresponds to a deep Gaussian process model when the first layer is treated as latent.

Given a new global input positions $x_{0}=\{x_{01},\ldots ,x_{0P}\}$, the hierarchy of the LGP that produces the global output prediction $Y_{0}$ can be seen as in Fig. 2.

Assume that the model parameters involved in ${\left\{GP_{1}^{\left(p\right)}\right\}}_{p=1,\ldots ,P}$ and $GP_{2}$ are known or estimated and we observe realizations $w_{p}$, w, and y of $W_{p}$, W and Y respectively given inputs $X_{p}=x_{p}$ for all p=1,…,P. Then, given that the one-dimensional outputs of ${\left\{GP_{1}^{\left(p\right)}\right\}}_{p=1,\ldots ,P}$ are conditionally independent, the posterior predictive distribution of the global output $Y_{0}$ at $x_{0}$ is given by $Y_{0}|x_{0},D$ with $D=\{y,w,w_{1},\ldots ,w_{P},x_{1},\ldots ,x_{P}\}$ and PDF $p(y_{0}|x_{0},D)$:


(3) $$\begin{array}{c} p(y_{0}|x_{0},D)=\int p(y_{0}|w_{0},y,w)p(w_{0}|x_{0},{\left\{w_{p},x_{p}\right\}}_{p=1,\ldots ,P})dw_{0}\\ =\int p(y_{0}|w_{0},y,w)\prod_{p=1}^{P}p(w_{0p}|x_{0p},w_{p},x_{p})dw_{0} \end{array}$$

where $p(y_{0}|w_{0},y,w)$ and $p(w_{0p}|x_{0p},w_{p},x_{p})$ are PDF’s of the posterior predictive distributions of $GP_{2}$ and $GP_{1}^{\left(p\right)}$, respectively; and $w_{0}=(w_{01},\ldots ,w_{0P})$. However, $p(y_{0}|x_{0},D)$ is analytically intractable because the integral in Equation (3) does not have a closed form expression because the GP posterior $p(y_{0}|w_{0},y,w)$ is Gaussian with both mean and variance that are nonlinear in $w_{0p}$, and marginalizing a Gaussian input $w_{0p}$ yields a non-Gaussian mixture with no closed-form density under standard kernels (e.g. squared-exponential, Matérn). However, the first two moments are available in closed form since they reduce to kernel expectations against a Gaussian. It can be shown that, given the GP specifications in Subsection Gaussian Processes, the mean, ${\tilde{\mu }}_{0}$, and variance, ${\tilde{\sigma }}_{0}^{2}$, of $Y_{0}|x_{0},D$ are expressed analytically as follows:


(4) $${\tilde{\mu }}_{0}=I{\left(x_{0}\right)}^{T}R{\left(w\right)}^{-1}y$$

(5) $$\begin{array}{cc} {\tilde{\sigma }}_{0}^{2}=y^{T}R{\left(w\right)}^{-1}J(x_{0})R{\left(w\right)}^{-1}y-{\left[I{\left(x_{0}\right)}^{T}R{\left(w\right)}^{-1}y\right]}^{2} & \\ +\sigma^{2}\left(1+\eta -\text{tr}\left[R{\left(w\right)}^{-1}J(x_{0})\right]\right) & \end{array}$$

where $I(x_{0})\in R^{M\times 1}$ with its *i*th element


(6) $$I_{i}=\prod_{p=1}^{P}E[k_{p}(W_{0p}(x_{0p}),w_{ip})];$$

$J(x_{0})\in R^{M\times M}$ with its *ij*-th element


(7) $$J_{ij}=\prod_{p=1}^{P}E[k_{p}(W_{0p}(x_{0p}),w_{ip})k_{p}(W_{0p}(x_{0p}),w_{jp})];$$

and the expectations in $I(x_{0})$ and $J(x_{0})$ have closed-form expressions under the linear kernel, squared exponential kernel, and a class of Matérn kernels (Titsias and Lawrence 2010, Kyzyurova *et al.* 2018, Ming and Guillas 2021). The linked GP is then defined as a Gaussian approximation $\hat{p}(y_{0}|x_{0},D)$ with its mean and variance given by ${\tilde{\mu }}_{0}$ and ${\tilde{\sigma }}_{0}^{2}$. Furthermore, the linked GP can be built iteratively to analytically approximate the posterior predictive distribution of outputs from any feed-forward GP systems. Research has shown that this approach provides an adequate approximation by minimizing the Kullback-Leibler divergence (Ming and Guillas 2021).

### 3.3 Deep Gaussian processes

An LGP model is useful when we have complete cases in the data. However, in many cases the data can have incomplete cases, e.g. although all input positions have their corresponding global output observed, some input locations can only have partially observed internal input/output, meaning that when we construct LGP between the global input and output, we must remove incomplete cases from the data to build individual GP models, leading to the loss of information. To address this issue, a deep GP (DGP) model can be used.

Consider *P* GP models $GP_{1}^{\left(p\right)}$ with *N* sets of *D*-dimensional input ($X\in R^{N\times D}$) and *N* sets of one-dimensional output ($W_{p}\in R^{N\times 1}$). Let $GP_{2}$ be another GP model with *N* sets of *P*-dimensional input ($W=[W_{1},\ldots ,W_{P}]\in R^{N\times P}$) and one-dimensional output ($Y\in R^{N\times 1}$). A two-layered DGP model is then created when we feed the predictions from ${\left\{GP_{1}^{\left(p\right)}\right\}}_{p=1,\ldots ,P}$ into the input of $GP_{2}$.

Note that if we have realizations x of X and y of Y fully observed while w of W are completely or partially missing, we need to use complete cases $\left[x_{o},w_{p,o}\right]$ in $\left[x,w_{p}\right]$ to train each $GP_{1}^{\left(p\right)}$, and complete cases $\left[w_{o},y_{o}\right]$ in [w,y] to train $GP_{2}$. This can be represented by the likelihood function:


(8) $$L_{\mathrm{LGP}}=p(y_{o}|w_{o})\prod_{p=1}^{P}p(w_{p,o}|x_{o}),$$

which leads to optimizations of individual GP likelihoods with complete cases.

However, for a two-layer DGP model, we can retain w and extract as much information as possible about its latent values from y, while jointly training the individual GPs by maximizing the likelihood function:


(9) $$L_{\mathrm{DGP}}=\int p(y|w)\prod_{p=1}^{P}p(w_{p}|x)dw_{p,m},$$

where p(y|w) is the multivariate Gaussian PDF of $GP_{2}$, $p(w_{p}|x)$ is the multivariate Gaussian PDF of $GP_{1}^{\left(p\right)}$, and $w_{p,m}$ is the missing cases (i.e. latent values) in $w_{p}$. However, due to the nonlinearity between y and w, the integral with respect to $w_{p,m}$ in Equation (9) is analytically intractable, making the inference of the DGPs challenging.

### 3.4 Deep GP with stochastic imputation

Recently, stochastic imputation (SI) was proposed to tackle the inference issue in deep GP (Ming *et al.* 2023), leveraging the fact that DGP and LGP are similar in structure. This approach provides a well-balanced trade-off between computational complexity and accuracy by combining the computational efficiency of variational inference (Salimbeni and Deisenroth 2017) and the accuracy of a full Bayesian approach (Sauer *et al.* 2023). The key concept of SI is converting a DGP emulator to multiple LGP emulators, each representing a DGP realization with imputed latent variables. Because some elements of the latent layer are observed, full imputation of the latent layer is not required.

Given a DGP hierarchy as described in Subsection Deep Gaussian Processes and realizations y of Y and $w_{p,o}$ of $W_{p,o}$, we can obtain point estimates of unknown model parameters in $GP_{1}^{\left(p\right)}$ for all p=1,…,P and $GP_{2}$, using the stochastic expectation maximization (SEM) algorithm (Ming *et al.* 2023). With the estimated model parameters, the DGP emulator gives the approximate posterior predictive mean and variance of $y_{0}(x_{0})$ at a new input position $x_{0}$ as described in Algorithm 1.

Algorithm 1. Construction of a two-layered DGP emulator

**Input:** (i) Realizations x, ${\left\{w_{p,o}\right\}}_{p=1,\ldots ,P}$, and y; (ii) A new input position $x_{0}$; (iii) The number of imputations *N*.

**Output**: Mean and variance of $y_{0}(x_{0})$.

1: **for**  i=1,…,N  **do** 

2:   Given x, ${\left\{w_{p,o}\right\}}_{p=1,\ldots ,P}$, and y, draw an imputation ${\left\{w_{p,m}^{i}\right\}}_{p=1,\ldots ,P}$ of the latent output ${\left\{W_{p,m}\right\}}_{p=1,\ldots ,P}$ via an Elliptical Slice Sampling (Nishihara *et al.* 2014) update.

3:   Construct the LGP emulator $LGP_{i}$ with the mean ${\tilde{\mu }}_{0,i}(x_{0})$ and variance ${\tilde{\sigma }}_{0,i}^{2}(x_{0})$, given x, y, and $\left\{w_{p}^{i}\right\}$ where $w_{p}^{i}=\{w_{p,o},w_{p,m}^{i}\}$.

4: **end for** 

5: Compute the mean $\mu (x_{0})$ and variance $\sigma^{2}(x_{0})$ of $y_{0}(x_{0})$ by

$$\begin{array}{cc} \mu (x_{0}) & =\frac{1}{N}\sum_{i=1}^{N}{\tilde{\mu }}_{0,i}(x_{0}),\\ \sigma^{2}(x_{0}) & =\frac{1}{N}\sum_{i=1}^{N}\left({\left[{\tilde{\mu }}_{0,i}(x_{0})\right]}^{2}+{\tilde{\sigma }}_{0,i}^{2}(x_{0})\right)-\mu {\left(x_{0}\right)}^{2}. \end{array}$$

One can extend Algorithm 1 to multi-layered DGPs with multi-dimensional outputs by applying the same algorithm and repeating step 2 for each layer. A detailed explanation of this generalization is provided in Ming *et al.* (2023).

### 3.5 Benchmarking

Our numerical experiments evaluated four different models to analyse the dataset.

### 3.6 Deep GP with stochastic imputation (DGP-SI)

We trained a unified deep GP model that integrated all components: it took timestamp as input, processed covariates in the latent layer, and predicted the output variable as output—all within a single end-to-end framework as described in Subsection Deep GP with Stochastic Imputation.

To set the baseline for our proposed models, we compared them with the following approaches:

1. Last-observation carried forward (LOCF) imputation: we used the last-known value of the variable until the next measurement is observed (Siddiqui and Ali 1998);
2. MICE: multiple imputation using chained equations (Jarrett *et al.* 2022)—ignoring the temporal dependency, relying only on the cross-sectional information between variables by treating each observation as independent and identically distributed (Rubin 1987, Little and Rubin 2019), and doing iterative imputation;
3. GP interpolation: we fitted a GP regressor with a squared exponential kernel individually for each covariate.

## 4 Numerical experiments

### 4.1 Clinical problem

Building on the Stewart-Fencl approach, the model was constructed with pH as the dependent variable and three independent variables: CO_2_ levels, strong ion differences (SID), and weak acid concentrations. As such, in this study, we developed a continuous pH estimation model based on the Stewart-Fencl approach (Stewart 1983). We then simulated real-world clinical scenarios by deliberately masking (intentionally withholding) portions of the covariates, mirroring the practical challenges of aligning laboratory test results with continuous bedside monitoring. Using measured pH levels to impute these missing covariate values, the aim was to provide clinicians with insights into patient status between laboratory tests.

The experiments were done on a dataset of 14 ICU admission windows selected at random from a paediatric intensive care unit. Each admission had a different number of data points, ranging from 19 to 115 hourly timestamps, and some patients had multiple admissions. To further de-identify the patients, the dates were shifted to future dates while retaining the time relationships.

To follow the physicochemical approach of acid-base balance, the model incorporated multiple blood gas measurements. Specifically, the model used partial carbon dioxide pressure (pCO_2_) and pH measurements and the difference between Na${\;}^{+}$ and Cl${\;}^{-}$ concentrations to represent the SID (Kellum 2000). Although CO_2_ levels can be measured through both blood gas analysis (Hassan and Martinez 2024) and capnography (ETCO_2_) (Raffe 2020), the numerical experiment was simplified by using only blood gas measurements.

For acid-base balance modelling, blood gas measurements are generally preferred over capnography for CO_2_ because they offer a more comprehensive and direct assessment of the body’s acid-base status (Anderson and Breen 2000, Lee *et al.* 2024). Additionally, the weak acid component was represented by lactate measurements from the blood gas analyser, as observations for albumin, phosphate, and urea were limited to only one or two measurements in half of the admission windows.

**Figure 3.**
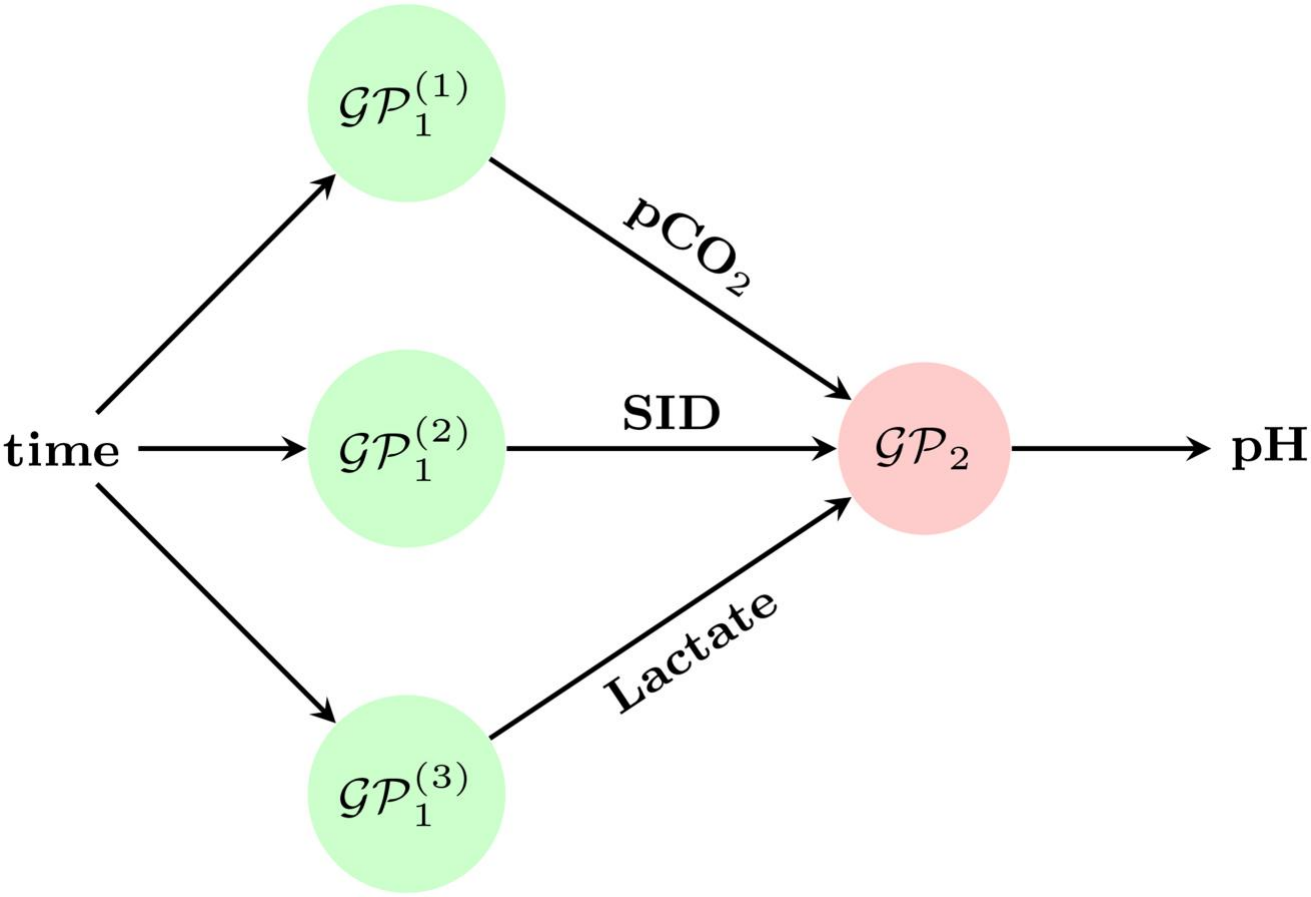
A two-layered deep Gaussian process (DGP) for pH prediction. This study compares two fitting approaches: individually fitting GP models by predicting the covariates using time as the input and the DGP with stochastic imputation (SI) method, where all the components are trained simultaneously.

The DGP-SI model was trained simultaneously for all components using the architecture illustrated in Fig. 3. As a baseline, an ablation study was also conducted using individually fitted GP models to predict the three covariates (pCO_2_, SID, and lactate) from time inputs. For a fair comparison, the MICE model only used time and pH data, excluding covariates since these would not be available during the inference process of our proposed models.

Following the literature, the data was preprocessed through several steps. First, the data was discretised into sequences of hourly intervals and the measurements were aggregated by taking the arithmetic means (Lipton *et al.* 2016, Ghosheh *et al.* 2024). Then, to evaluate the model’s robustness, either the observed pH or the covariates were randomly masked with varying proportions (10%, 20%, 30%, and 40%) (Beaulieu-Jones and Moore 2017, Jafrasteh *et al.* 2023). Realistically, the proportion of missingness typically ranges from about 15% to 30% according to a similar study where a GP model was also used as a benchmark (Luo *et al.* 2018). To evaluate the robustness of our approach under more challenging conditions, we further conducted stress tests at 40% missingness. Finally, z-score transformation was applied to both pH values and the covariates to standardize the data distribution.

To evaluate model performance given the presence of measurement noise, the accuracy of missing value imputations was defined as the mean absolute error (MAE):


(10) $$\text{MAE}=\frac{1}{N\times D}\sum_{i=1}^{N}\sum_{d\in D}|Y_{id}-{\hat{Y}}_{id}|$$

where *N* is the total number of missing values being evaluated, $Y_{id}$ is the *i*-th true value that was masked and ${\hat{Y}}_{id}$ is the *i*-th estimated value at dimension *d*. In the GP-based methods, the model prediction is computed as the mean of the predictive distribution.

Additionally, the models were evaluated with respect to uncertainty quantification using the negative log likelihood (NLL), defined as:


(11) $$\text{NLL}=-\sum_{i=1}^{N}\;\text{log}\;p(Y_{i}|X_{i};\theta )$$

where $Y_{i}$ denotes the *i*-th masked true value, and $X_{i}$ represents the input to the probability density function $p(Y_{i}|X_{i})$, parametrized by the learnable parameters θ. Note that, since the LOCF method does not provide uncertainty quantification, it was excluded from this evaluation.

## 5 Results

### 5.1 Imputing missing values in covariates

The first experiment was to predict missing values with varying proportions from three variables: pCO_2_, SID, and lactate as the weak acid. To ensure comparable results, all models were standardized to use time as the input variable and pH as the target variable. All three variables were measured at the same time as pH. This experiment used all the observed pH values to link the three covariates as suggested by the Stewart-Fencl physicochemical approach. Since training a linked GP with sequential design, in this case, was just individually fitted GPs with complete case analysis, there were only four models to compare in this experiment.

**Figure 4.**
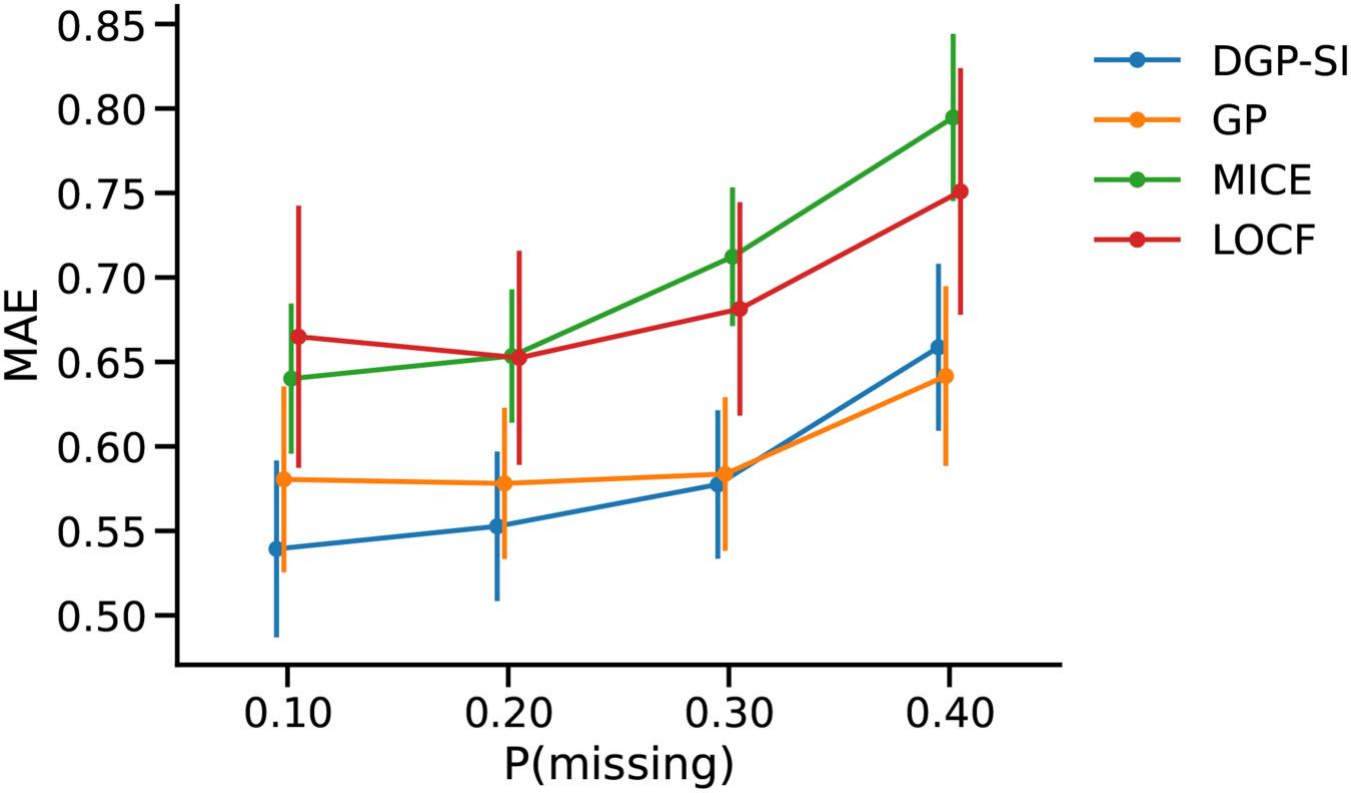
Average MAE values in imputing the covariates. The error bars represent the standard error of the MAE values across all admission windows. DGP, which combines longitudinal and cross-sectional information, performs better in imputing missing values in the covariates, particularly at lower missingness levels. However, as the proportion of missing values increases, methods that rely on longitudinal information become more effective.

The performance comparison revealed that DGP achieved the lowest error rates at 10% and 20% missing values, performed similarly to GP at 30%, and slightly underperformed compared to GP at 40% (Fig. 4). However, as noted earlier, a missingness rate of 40% is only used here as a benchmark and is not typical in clinical settings. The proposed method works best when the amount of missing data is at realistic, clinically expected levels. Both DGP and GP demonstrated better performance than MICE and LOCF, with LOCF showing lower error rates than MICE as missingness increased. These findings suggest that longitudinal information is more valuable than cross-sectional information for covariate imputation. However, DGP’s low error rates indicate that combining both longitudinal and cross-sectional information yields optimal results.

Given the results, practitioners should generally start with a GP model when the underlying data is expected to be smooth or can be well-approximated using established kernels. If the validation metrics indicate that a higher model capacity is needed and a hierarchical structure aligns with the mathematical formulation of the problem, e.g. the physicochemical modelling, then using DGP may be appropriate. In our case, DGP helps to address the partial information issues that we have in the covariates, as well as the non-stationarity. However, it is important to note that adding more layers to DGPs may provide little improvement in accuracy, and past a certain depth, the additional computational cost outweighs the marginal gains in performance (Dunlop *et al.* 2018, Ming *et al.* 2023).

### 5.2 Uncertainty quantification

**Figure 5.**
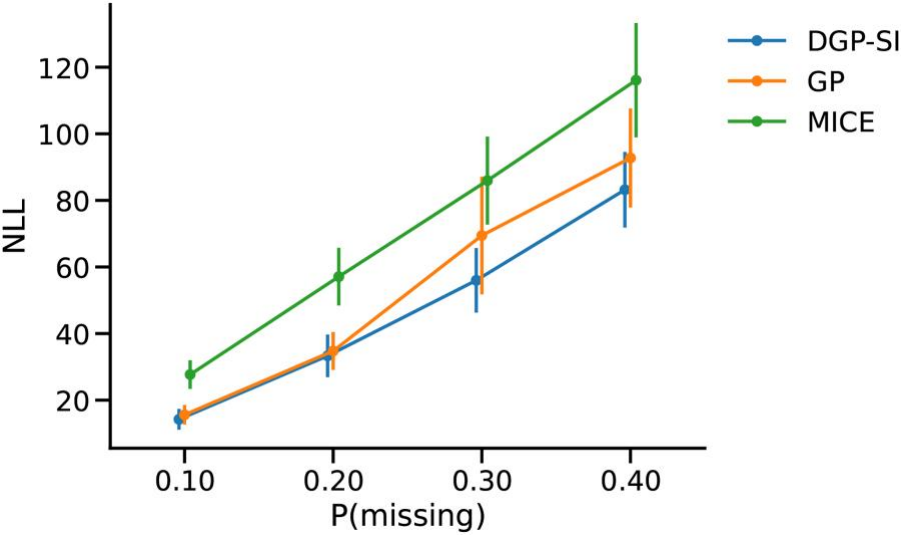
Average NLL values in imputing the covariates. The error bars represent the standard error of the NLL values across all admission windows. DGP generally performs better at imputing missing values whilst maintaining tighter uncertainty bounds for the covariates. As the proportion of missing values increases, all models show higher NLL values.

When incorporating uncertainty quantification through NLL evaluation, the results show that all models exhibited higher NLL values as the proportion of missing data increased (Fig. 5). Furthermore, although DGP and GP achieved comparable NLL at 10% and 20% missingness, DGP maintained tighter uncertainty bounds than GP at 30% and 40%, resulting in lower NLL. Consistent with the MAE results, both DGP and GP outperformed MICE, whilst the last-known imputation method was excluded from this evaluation as it does not provide uncertainty quantification.

**Figure 6.**
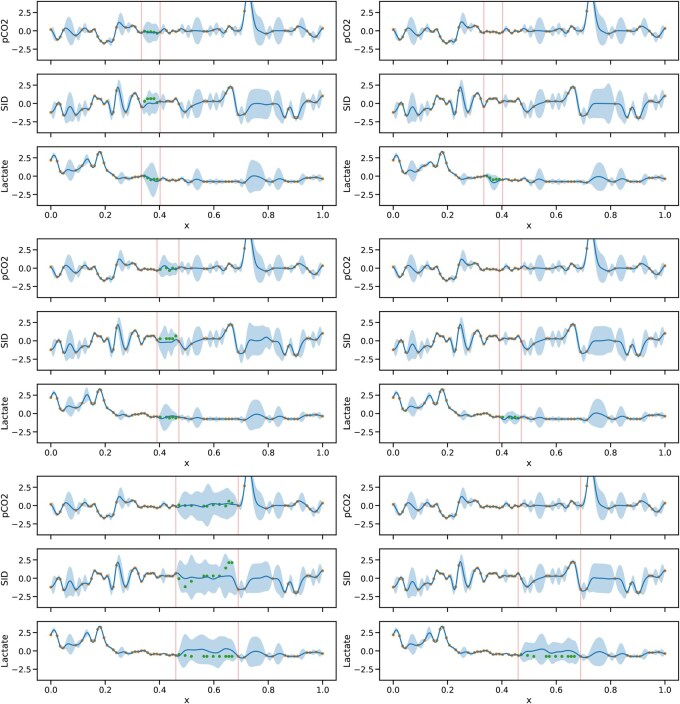
Uncertainty quantification derived from the DGP-SI model when imputing missing values in the covariates. The dots represent observed values and the dots between the two vertical lines show masked values. The *x*-axis shows scaled time, and the *y*-axis shows standardized covariate values. The uncertainty for lactate resulting from manually masking the three covariates (left) is greater than that from only masking lactate (right).

As the covariates were connected through pH in the output layer using DGP-SI, an observation from one covariate could affect the uncertainty of another covariate where an observation was unavailable. To demonstrate this effect, differences in uncertainty were compared by manually masking observations from the three covariates in three different intervals, focusing on masking lactate within these intervals. The experiment revealed that the uncertainty in lactate, shown in Fig. 6, was less when only the observed points in lactate were masked instead of all three covariates being masked.

Given that the number of tests performed on a patient is often restricted, particularly when procedures are invasive or carry associated risks, clinicians face the challenge of obtaining clinical insight from limited measurements. As demonstrated in Fig. 6, DGP-SI offers an advantage in such situations by enabling the integration of longitudinal patterns within a specific covariate alongside cross-sectional information from the remaining covariates. This approach can support informed test selection and imputation strategies, optimizing both patient safety and diagnostic yield.

## 6 Discussion

This paper demonstrated that DGP-SI, initially developed for uncertainty quantification in computationally expensive models, could effectively handle missing values in critical care data from different sources. The analysis across 14 admission windows showed that DGP-SI performed better in imputing missing covariate values, particularly when the proportion of missing data was low. This approach offers clinicians insight into patient states between measurements whilst providing uncertainty quantification, hence attaching a measure of confidence that addresses the inherent uncertainties in medical science (Cabitza *et al.* 2017).

A similar approach for inferring missing values in time series, such as Variational GP Dynamical Systems (Damianou *et al.* 2011), can be found in the literature. Although Variational GP and DGP-SI share similar aims–namely, modelling high-dimensional sequential data and providing uncertainty quantification–we chose to use the Deep Gaussian Process with Stochastic Imputation (Ming *et al.* 2023) due to its favourable properties in uncertainty quantification. A common concern with the variational inference used in Variational GP Dynamical Systems (Damianou *et al.* 2011) is that it may fail to capture important aspects of posterior uncertainty. Additionally, maximization of the ELBO can be challenging due to non-convexity or the large number of model parameters introduced by the layered structure of GPs (Ming *et al.* 2023). As an alternative, Ming *et al.* (2023) showed that using Elliptical Slice Sampling (ESS) within a Gibbs sampler (ESS-within-Gibbs) to impute latent variables in DGPs could lead to better uncertainty quantification.

From a clinical perspective, accurately imputing missing values with quantified uncertainty can impact decision-making, especially in time-sensitive critical care scenarios. Clinicians often face the challenge of making treatment decisions with incomplete data, and the uncertainty quantification provided by DGP-SI can shape how they are interpreted and applied in clinical workflows, e.g. in guiding decisions on whether to order additional tests or cautioning against over-reliance on imputed results. Rather than serving solely as a methodological complication, the resulting risk distributions can provide valuable insights to support clinical decision-making and facilitate conversations with patients and their families (Mathiszig-Lee *et al.* 2022). Additionally, this method has the potential to enhance early warning systems in intensive care units, identifying deteriorating patients earlier while reducing false alarms.

The proposed approach also poses challenges for future work with two main limitations. First, it is less effective in emulating the Stewart-Fencl physicochemical model for pH prediction, likely due to error propagation through intermediate variables. Second, as highlighted in Ming *et al.* (2023), the method becomes computationally expensive with larger datasets. To handle this, the data was partitioned into shorter admission windows. We did not compare the results with the non-discretised time data for two reasons. First, the absolute number of missing values would differ when using non-discretised raw data, even with the same missingness proportion, complicating fair comparison. Second, the discretization step aligns the data with clinically relevant time intervals, reflecting the practical realities of ICU monitoring where clinicians are not always available at the bedside, thus only observing the aggregated measurements. Note that such an approach may not be feasible in settings with high-frequency measurements, such as those from wearable devices.

To address these limitations, several paths forward exist. The computational burden can be reduced through time discretization and data aggregation, as demonstrated in this work. Alternative solutions include implementing sparse GP approximations (Snelson and Ghahramani, 2007, Bauer *et al.* 2016) or utilizing GPU parallelization for exact GP computations (Wang *et al.* 2019). Additionally, further research should evaluate the model’s performance on multimodal datasets with naturally varying patterns of missing data, focusing on scenarios where sufficient observations exist to be emulated using a deep GP model.

## Data Availability

The data underlying this article will be shared on reasonable request to the corresponding author.
